# Study of Polyethylene Oxide-*b*-Poly(*ε*-caprolactone-*ran*-*δ*-valerolactone) Amphiphilic Architectures and Their Effects on Self-Assembly as a Drug Carrier

**DOI:** 10.3390/polym17081030

**Published:** 2025-04-10

**Authors:** Chaoqun Wang, Tong Wu, Yidi Li, Jie Liu, Yanshai Wang, Kefeng Wang, Yang Li, Xuefei Leng

**Affiliations:** 1 State Key Laboratory of Fine Chemicals, Liaoning Key Laboratory of Polymer Science and Engineering, Department of Polymer Science and Engineering, School of Chemical Engineering, Dalian University of Technology, Dalian 116024, China; wangchaoqun@mail.dlut.edu.cn (C.W.); liyidi0926@163.com (Y.L.); 18604090664@dlut.edu.cn (J.L.); wangyanshai@dlut.edu.cn (Y.W.); wangkf@dlut.edu.cn (K.W.); 2 SINOPEC Ningbo New Materials Research Institute Company Limited, Ningbo 315000, China; wut039.zhlh@sinopec.com

**Keywords:** amphiphilic copolymers, drug delivery, architectures, self-assembly

## Abstract

Amphiphilic block copolymers with complex topologies (e.g., star and brush topologies) have attracted significant attention in drug delivery owing to their superior performance over linear micelles. However, their precise synthesis and structure–property relationships require further investigation. In this study, hydroxylated polybutadiene with adjustable topology and hydroxyl group density was employed as a macroinitiator to synthesize well-defined amphiphilic poly (ethylene oxide)-*b*-poly(*ε*-caprolactone-*ran*-*δ*-valerolactone) (PEO-*b*-P(CL-*ran*-VL)) copolymers via ring-opening polymerization (ROP). A series of linear, star, linear–comb, and star–comb copolymers were prepared as curcumin-loaded micellar carriers for the study. The self-assembly behavior, drug encapsulation efficiency, and in vitro release profiles of these copolymers in aqueous environments were systematically investigated. The results demonstrated that increasing the branch length of star–comb copolymers effectively reduced micelle size from 143 to 96 nm and enhanced drug encapsulation efficiency from 27.3% to 39.8%. Notably, the star–comb architecture exhibited 1.2-fold higher curcumin encapsulation efficiency than the linear counterparts. Furthermore, the optimized star–comb nanoparticles displayed sustained release kinetics (73.38% release over 15 days), outperforming conventional linear micelles. This study establishes a quantitative structure–property relationship between copolymer topology and drug delivery performance, providing a molecular design platform for programmable nanocarriers tailored to diverse therapeutic requirements of various diseases.

## 1. Introduction

Biocompatible copolymers with complex, well-defined macromolecular architectures are essential [1,2,3]. The effectiveness of drug-loaded micelles is determined by the structural features of their component amphiphilic block copolymers [4]. Linear [5,6,7,8], cyclic [9,10,11,12], comb [13,14,15], star [16,17,18], and complex architectures [19,20] have been investigated as nanomaterials for drug delivery. Numerous studies have indicated that the molecular structure substantially influences micelle characteristics, including encapsulation efficiency, release kinetics, and drug loading capacity [4,21,22,23]. Thus, further research is needed to fine-tune polymer structures and create optimal nano delivery systems for specific biomedical applications.

Star-shaped polymers with multiple arms exhibit enhanced drug-loading capacities compared to their linear counterparts, whereas comb-like architectures offer better micelle stability [24,25]. Star–comb copolymers combine the characteristics of star- and comb-shaped copolymers, exhibiting a higher core density and lower critical micelle concentration (CMC) [26]. Although the overall performance of the star–comb structure is better than that of a single structure, having too many arms may negatively affect the performance. A recent study demonstrated that the 4-arm star–comb PCL_10_-*b*-PEG_20_ exhibited 1.6-fold higher drug loading (DL) than its 10-arm counterpart under identical conditions [19], likely due to reduced steric hindrance, which promotes drug nanophase formation. In addition, the relationship between the hyperbranched copolymers and particle size is not controlled by the molecular weight alone but may also be influenced by the architecture. The topological architecture of copolymers is a critical feature for designing efficient nanocarriers for drug delivery [20]. By meticulously regulating the structural parameters of copolymer micelles, including morphology, molecular weight, and the hydrophilic-hydrophobic ratio, it was possible to achieve tailored micelle performance [26,27,28]. Despite extensive investigations of star and comb architectures, the potential of highly branched amphiphilic block copolymers with complex architectures remains unexplored.

Amphiphilic copolymers composed of PEG and PCL are typical drug carrier materials. PCL is hydrophobic and biocompatible, and PEG is hydrophilic and can avoid immune recognition. These properties make PEG-PCL copolymers ideal drug carriers [29,30]. However, poly (caprolactone) (PCL) homopolymers have high crystallinity and hydrophobicity, resulting in long hydrolytic degradation times, limiting their application in the drug field [31,32]. The copolymerization of caprolactone with flexible monomers (such as *δ*-valerolactone) not only accelerates degradation but also enables the design of hyperbranched star–comb architectures with tunable crystallinity, which is critical for balancing drug release kinetics and carrier stability [33,34,35].

The synthesis of star–comb PCL-*b*-PEG copolymers mostly depends on a combination of multiple polymerization methods, such as ring-opening polymerization (ROP), atom transfer polymerization (ATRP), and coupling reactions. Although these techniques can be employed to create copolymers with desired molecular weights and precisely controlled topological structures, the complex synthesis steps are challenging. To understand the structure–property relationships, a reliable synthetic strategy is required. Recently, our group established an effective grafting strategy to easily obtain hyperbranched copolymers with adjustable molecular weights and comb densities using hydroxylated polybutadiene with controllable hydroxyl groups as macroinitiators [36,37,38,39].

In this study, a faster method for synthesizing polymers with complex molecular architectures is proposed. This method utilizes the controllable number of hydroxyl groups and the designable molecular architecture of hydroxylated polybutadiene. A series of amphiphilic polyethylene oxide-*b*-poly (*ε*-caprolactone-*ran*-*δ*-valerolactone) (PEO-*b*-P(CL-*ran*-VL)) copolymers with different branch lengths were synthesized through continuous ring-opening polymerization. In order to assess the structure–activity relationship between structural parameters and the efficacy of drug-loaded micelles, a comprehensive library of copolymers with linear, star, linear–comb, and star–comb configurations was developed. These copolymers were used as drug-loaded micelles, with curcumin as the drug model. The relationship between structural parameters (branch length) and micelle size, self-assembly behavior in aqueous solution, drug-loading capacity, and in vitro release was studied in detail. Cell compatibility tests were conducted on micelles with various structures. PANC-1 cells (an epithelioid carcinoma cell line) were used as target cells to assess the therapeutic potential of the micelles for tumor cells.

## 2. Materials and Methods

### 2.1. Materials

Butadiene (Dalian Special Gases Co., Ltd. (Dalian, China)), trifluoromethanesulfonic acid (TfOH, 98%, Aladdin (Shanghai, China)), hydrogen peroxide (H_2_O_2_, 30 wt%, Aladdin (Shanghai, China)), t-BuP_4_ (0.8 M, Sigma-Aldrich (St. Louis, MO, USA)), benzyl alcohol ((BnOH), Aladdin (Shanghai, China)), pentaerythritol (C_5_H_12_O_4_, 98%, Aladdin (Shanghai, China)), formic acid (HCOOH, 88% Aladdin (Shanghai, China)), stannous octanoate (Sn(Oct)_2_, 97%, Sigma-Aldrich (St. Louis, MO, USA)), *ε*-caprolactone (*ε*-CL, 99%, Sigma-Aldrich (St. Louis, MO, USA)), and *δ*-valerolactone (*δ*-VL, 99%, Sigma-Aldrich (St. Louis, MO, USA)) were used as received. Ethylene oxide (EO, 99.5%, Sigma-Aldrich (St. Louis, MO, USA)). Curcumin (Cur; 99.5%, Sigma-Aldrich (St. Louis, MO, USA)) and Cell Counting Kit-8 (CCK-8) were obtained from Solarbio Life Sciences (Beijing, China). n-Butyllithium ((n-BuLi), 1.4 M, Aladdin (Shanghai, China)) was used to remove the inhibitors of butadiene and ethylene oxide (EO, 99.5%, Sigma-Aldrich (St. Louis, MO, USA)).

### 2.2. Cell Lines

PANC-1 cells, an epithelioid carcinoma cell line derived from humans, were obtained from the Shanghai Institute of Biochemistry and Cell Biology, Chinese Academy of Sciences (Shanghai, China).

### 2.3. Synthesis of PEO-b-P(CL-ran-VL)

All experiments and reactions were conducted in an argon environment. The flask was desiccated using three cycles of flaming, argon purging, and evacuation.

#### 2.3.1. Synthesis of Linear and Star Hydroxylated Polybutadiene

Linear- and star-shaped hydroxylated polybutadiene (L-PB-OH and S-PB-OH) were synthesized as macromolecular initiators according to previously reported methods [39,40].

Linear and star hydroxylated polybutadienes were synthesized via anionic polymerization. First, the butadiene monomer was purified twice using n-BuLi. The flasks were then sealed and weighed. Butadiene was introduced into the flask via a syringe using a vapor-liquid method. The flask was weighed again. Hexane was added to make a solution (1:8 butadiene-to-hexane by mass). To initiate polymerization, n-BuLi was added to the reaction flask, and the reaction was conducted at 50 °C for 3.5 h. Subsequently, isopropanol was introduced to quench the reaction mixture. The reaction solution was then washed with deionized water until it reached a neutral pH. Following solvent removal, the product was dried to a constant weight to obtain L-PB (yield = 98%).

**Scheme 1:**
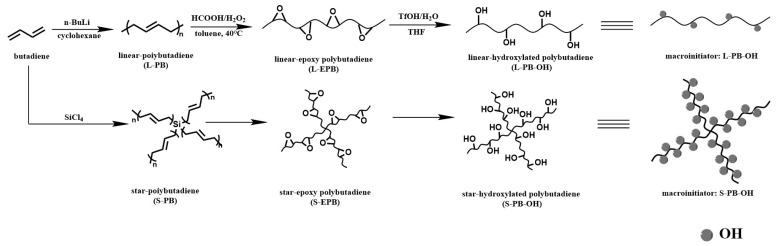
Synthesis of macroinitiator.

Star polybutadiene (S-PB) was synthesized in a manner analogous to linear polybutadiene, with the distinction being the addition of a stoichiometric amount of SiCl_4_ coupling agent prior to termination. The polymerization conditions for L-/S-PB are shown in Scheme 1.

The preparation method for star-shaped epoxy polybutadiene (S-EPB) was the same as that for linear epoxy polybutadiene(L-EPB). Polybutadiene (8.00 g), vacuum-dried, and toluene (200 mL) were introduced into the flask. Toluene was added to the polybutadiene to obtain a homogeneous solution. Then, formic acid (2.78 g) and H_2_O_2_ (6.72 g, 30% concentration) were added to the solution, and the mixture was reacted at 40 °C for 2 h. Finally, the solvent was removed, and the product was dried. The final product obtained was epoxy polybutadiene (L-EPB) with a yield of 93%.

The preparation method for star-shaped hydroxylated polybutadiene (S-PB-OH) was the same as that for linear hydroxylated polybutadiene (L-PB-OH). Vacuum-dried EPB (8.00 g) was dissolved in 115 mL of THF to form a homogeneous solution. Then, trifluoromethanesulfonic acid (TfOH, 6.46 g) and deionized water (7.74 g) were added to the solution, stirred thoroughly, and transferred to a separatory funnel for extraction with CHCl_3_. After removing the solvent and drying, L-PB-OH was obtained with a yield of 92%.

The Supplementary Data contain detailed descriptions of the macroinitiators listed in Table S1. The syntheses of L-/S-PB-OH are shown in Scheme 1.

#### 2.3.2. Synthesis of Linear–Comb and Star–Comb Polyethylene Oxide (LC-/SC-PEO)

LC-/SC-PEO was synthesized according to a previously reported procedure [36]. In brief, the synthesis process commenced with the preparation of a dry THF solution containing t-BuP_4_ (0.42 mL, 0.32 mmol) and L-PB-OH (0.2 g, 1.66 mmol OH). Specifically, t-BuP_4_ and L-PB-OH were dissolved in dry THF (40 mL) at a ratio of [–OH]/[t-BuP_4_] = 1/0.2. The EO solution (10.6 mL, 82.5 mmol, [EO]_0_ = 15.52 M) was slowly injected into the reaction flask using a syringe to initiate the reaction. The flask was then placed in a water bath, heated to 45 °C, and stirred continuously for 48 h to facilitate the reaction to occur. Upon completion of the reaction, the mixture was quenched by the addition of AcOH (1 mL) and precipitated with n-hexane. Finally, the product was collected and dried under vacuum to obtain LC-PEO with a yield of 93%.

The synthesis of SC-PEO followed a procedure similar to that of the LC-PEO synthesis. The primary difference was the use of S-PB-OH as the macroinitiator instead of L-PB-OH.

**Scheme 2:**
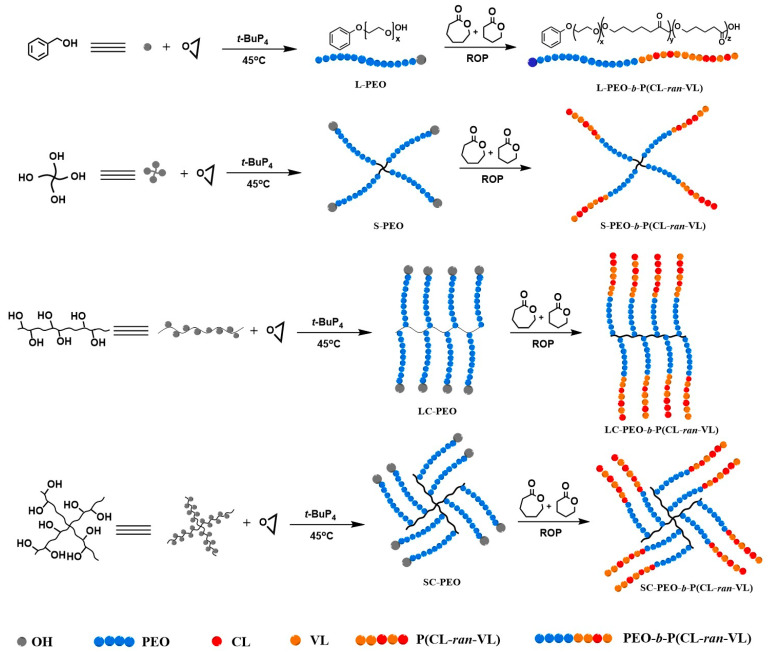
Synthesis of PEO-*b*-P(CL-*ran*-VL) with different architectures.

This substitution was the key variation in the synthesis process, and the polymerization conditions of SC-PEO are shown in Scheme 2.

#### 2.3.3. Synthesis of Linear–Comb and Star–Comb Polyethylene Oxide-b-Poly(ε-caprolactone-*ran-δ*-valerolactone) (LC-/SC-PEO-*b*-P(CL-*ran*-VL))

LC-PEO-*b*-P(CL-*ran*-VL): LC-PEO (2.0 g), *ε*-CL (1.6 g), *δ*-VL (0.4 g), and Sn(Oct)_2_ (7.0 mg) were sequentially added to a flask under an arg. The mixture was then stirred for 15 h at 120 °C. The product was settled from n-hexane and dried in a vacuum oven until a constant weight was achieved. The synthesis of SC-PEO-*b*-P(CL-*ran*-VL) was similar to that of LC-PEO-*b*-P (CL-*ran*-VL), except that SC-PEO was used instead of LC-PEO.

#### 2.3.4. Synthesis of Linear and Star Polyethylene Oxide-b-Poly(ε-caprolactone-*ran*-*δ*-valerolactone) (L-/S-PEO-*b*-P(CL-*ran*-VL))

To synthesize the L-/S-PEO-*b*-P(CL-*ran*-VL) precursors (L-/S-PEO), BnOH (0.11 mL, 1.02 mmol), t-BuP_4_ (0.26 mL, 0.2 mmol), and EO solution (5.4 mL, 41.24 mmol) were added to the flasks, which were then sealed and incubated at 45 °C for 48 h. The resulting compound was solubilized in CHCl_2_ and precipitated using n-hexane. The copolymer was then dried to a constant weight. The synthesis of S-PEO was similar to that of L-PEO, except that pentaerythritol was used instead of benzyl alcohol.

The synthesis of L-/S-PEO-*b*-P(CL-*ran*-VL) was similar to LC-/SC-PEO-*b*-P(CL-*ran*-VL), except that L-/S-PEO was present instead of LC-/SC-PEO.

The syntheses of the linear, star-shaped, linear–comb, and star–comb amphiphilic block copolymers, PEO-*b*-P(CL-*ran*-VL), are shown in Scheme 2.

### 2.4. Preparation of Polymer Micelles

To prepare blank copolymer micelles [17], a block copolymer (5.0 mg) was dissolved in THF (2 mL) and stirred vigorously to obtain a homogeneous suspension. The suspension was added to distilled water, and THF was removed by evaporation at room temperature to obtain polymer micelles with a concentration of 0.5 mg/mL.

To prepare curcumin-loaded copolymer micelles [17,19], 5.0 mg of the amphiphilic block copolymer and 1.0 mg of curcumin were dissolved in 2 mL of tetrahydrofuran. The mixture was gradually introduced into 10 mL of deionized water while continuously stirring. After removing the THF by evaporation, the micelle dispersion was further diluted with deionized water. Curcumin-encapsulated polymer micelles were separated using 0.45 μm membrane filtration to eliminate unadulterated curcumin aggregates.

### 2.5. Characterization

#### 2.5.1. Polymer Characterization

The molecular weights and distributions of the obtained polymers were determined by gel permeation chromatography (GPC) using a Waters 1515 HPLC pump and a Waters 2414 refractive index detector(Waters Corporation, Milford, MA, USA). THF was used as the eluent at a flow rate of 1.0 mL/min. The calculation was performed using polystyrene (1.2 × 10^3^ to 2.6 × 10^6^ g/mol) as the standard.

A Bruker Advance 400 MHz spectrometer (Bruker Technology Company, Fällanden, Switzerland) was used for the ^1^H NMR analysis at room temperature in deuterated solvents with Si(CH_3_)_4_ as an internal standard.

#### 2.5.2. The Shape and Size of Micelles

The morphology of the PEO-*b*-P(CL-*ran*-VL) micelles was examined using transmission electron microscopy (TEM, HT7700, HITACHI, Tokyo, Japan). To prepare the specimens for TEM, a drop of micelle solution with a concentration of 0.5 mg/mL was diluted fivefold and deposited onto a carbon-coated copper grid and then air-dried. The dilution was intended to reduce the aggregation of the sample during drying [29].

The particle size distribution and average hydrodynamic diameter were determined using dynamic light scattering measurements (Malvern Zetasizer Nano ZS90, Malvern, UK), and the compounds were tested at a concentration of 0.5 mg/mL in pure water at 25 °C.

#### 2.5.3. Critical Micelle Concentration Determination (CMC)

Fluorescence spectroscopy (JASCO FP-6500, JASCO, Tokyo, Japan) was used to measure the CMC of the PEO-*b*-P (CL-*ran*-VL) micelles using pyrene as a fluorescent molecular probe. The excitation spectra of pyrene fluorescence were measured at various micelle concentrations.

The fluorescence emission spectra of micelle solutions with pyrene concentrations ranging from 10 × 10^−1^ to 10 × 10^−4^ mg/mL were recorded while keeping the pyrene concentration constant at 1.0 × 10^−6^ mol/L. Spectral intensity ratios (I_339_/I_384_) were plotted against logarithmic polymer concentrations (lgC), with the CMC identified as the intersection point of the regression curves [41].

### 2.6. Drug Loading and Encapsulation Efficiency

To quantify curcumin loading and encapsulation efficiency, a UV-Vis absorption standard curve for curcumin was first prepared to establish a linear relationship between curcumin concentration and absorbance (Figure S1). The drug-loaded micelles were dissolved in a PBS/acetone mixture (4:1, *v*/*v*). Acetone ensured the complete solubilization of curcumin. The UV-Vis absorption spectrum of the solution was measured, and the absorbance peak was recorded to determine the concentration of curcumin. The mass of the drug encapsulated in the micelles was calculated. The specific calculation method and steps can be obtained from the Supplementary Information. The drug loading capacity (DL%) and encapsulation efficiency (EE%) were calculated using Equations (1) and (2), respectively, written as follows:

(1) $$DL\left(\%\right)=\frac{mass\;of\;drug\;encapsulated\;in\;micelles}{mass\;of\;micelles\;containing\;drug}\times 100\%$$

(2) $$EE\left(\%\right)=\frac{mass\;of\;drug\;encapsulated\;in\;micelles}{mass\;of\;drug\;in\;feed}\times 100\%$$

### 2.7. In Vitro Release

Study on in vitro drug release using a UV–visible spectrophotometer, determine the concentration of curcumin in the solution through the UV absorption standard curve of curcumin, and convert it to the concentration of curcumin in the release medium to calculate the cumulative release of curcumin.

The micelles were diluted with pure water to obtain a curcumin-loaded copolymer micelle solution with a concentration of 0.6 mg/mL. The total curcumin content in the initial drug-loaded polymer micelles was 0.8 mg. At 37 °C, 20 mL of PBS (pH 7.4) containing 0.1% Tween 80 and 0.0015% ascorbic acid was used as the release medium, and 8 mL of curcumin-loaded copolymer micelle solution was dialyzed (MWCO 1000 Da) in the release medium. The release medium was removed at a specific time, and 20 mL of fresh release medium was added.

To accurately determine the concentration, acetone (25% *v*/*v*) was added to ensure complete dissolution of curcumin. The peak value was recorded at 427 nm using a UV-Vis spectrophotometer, and the cumulative release percentages (Q%) were calculated using Equation (3), written as follows [42,43,44]:

(3) $$Q\left(\%\right)=\frac{V\times \sum_{n=1}^{n=t-1}C_{t}}{W}\times 100\%$$

V: Release medium volume (20.0 mL);

C_t_: Concentration of curcumin at specific time point t (mg/mL);

W: Total curcumin content in the initial drug-loaded polymer micelles.

### 2.8. Cytotoxicity Study

The CCK-8 method was employed to assess the cytotoxicity of polymer micelles in PANC-1 pancreatic cancer cells [45,46,47]. PANC-1 cells in the logarithmic phase were seeded in 96-well plates (5 × 10^4^ cells/well) and maintained for 24 h. Subsequent treatment involved exposure to either blank micelles (1.0 mg/mL) or drug-loaded formulations (50 μg/mL) for 48 h. Following CCK-8 reagent addition (100 μL/well) and 4-h incubation, we measured optical density (OD) at 450 nm using microplate spectroscopy. Cell viability calculations were performed using Equation (4), written as follows [48]:

(4) $$\mathrm{Cell}\;\mathrm{viability}(\%)=\frac{{\mathrm{OD}}_{\mathrm{sample}}-{\mathrm{OD}}_{\mathrm{blank}}}{{\mathrm{OD}}_{\mathrm{control}}-{\mathrm{OD}}_{\mathrm{blank}}}\times 100\%$$

OD_sample_: OD density of the polymer micelle sample.

OD_blank_ and OD_control_ denote the optical density measurements for the blank and control groups, respectively.

## 3. Results and Discussion

### 3.1. Polymer Design

L-/S-PEO was synthesized using Bn(OH) or pentaerythritol as initiators and t-BuP_4_ as a catalyst via the ROP of ethylene oxide to obtain linear or star copolymers. Subsequently, L-/S-PEO-*b*-P(CL-*ran*-VL) was synthesized using L-/S-PEO as a macroinitiator via ROP of *ε*-CL and δ-VL.

According to previous research, a polymer with an *ε*-CL/*δ*-VL ratio of 8:2 exhibits optimal performance [39]. For each architectural configuration, copolymers were designed with a 1:1 ratio of PEO and P(CL-*ran*-VL) blocks to explore the effect of side-chain length on micelle performance. L_x_-/S_x_-PEO-b-P (CL-ran-VL) copolymers with different side chain lengths were synthesized, where x represents the relative extent of the side chain length, with x = 1 indicating the minimum length and x = 3 indicating the maximum length of the side chain.

To obtain LC-/SC-PEO-*b*-P(CL-*ran*-VL), LC-/SC-PEO was initially synthesized using L-PB-OH or S-PB-OH as macroinitiators via the ROP of ethylene oxide. To achieve an equivalent branching degree, the number of hydroxyl groups (N_OH_) per molecule in both L-PB-OH and S-PB-OH was set to 23 (Table S1). Subsequently, LC-/SC-PEO-*b*-P(CL-*ran*-VL) was synthesized using LC-/SC-PEO as a macroinitiator via ROP of *ε*-CL and *δ*-VL. The general formula of linear–comb or star–comb copolymers was LC_x_-/SC_x_-PEO-*b*-P(CL-*ran*-VL), where x represents the relative size of the side chain length.

The microstructures of L-/S-/LC-/SC-PEO-*b*-P(CL-*ran*-VL) were analyzed using GPC and ^1^H NMR. Table 1 summarizes the polymerization results. All copolymers achieved the expected degree of polymerization, with a polydispersity coefficient of less than 1.21, indicating a relatively narrow molecular weight distribution.

**Figure 1:**
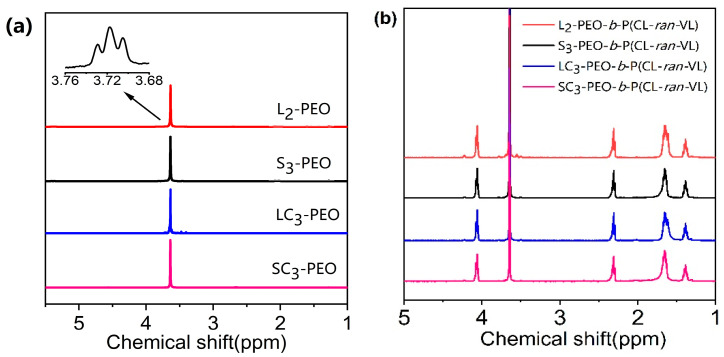
^1^H NMR spectra of (**a**) PEO and (**b**) PEO-*b*-P(CL-*ran*-VL) in CDCl_3_.

Figure 1 shows the ^1^H NMR spectra of PEO-*b*-P(CL-*ran*-VL) with different topologies. The C-H bond of PEO was observed at *δ* = 3.64 ppm, indicating the successful synthesis of PEO (Figure 1a). Subsequent to P(CL-*ran*-VL) blocking, the ^1^H NMR spectra of the CL or VL units overlapped at *δ* = 1.55–1.75 ppm, *δ* = 2.31–2.35 ppm, and *δ* = 4.05–4.10 ppm [39]. The characteristic peak of CL was observed at 1.38 ppm. The peak at *δ* = 3.6 ppm indicates the presence of the PEO main chain, confirming the successful synthesis of the copolymer (Figure 1b). The hydrophilic and hydrophobic block ratios of the block copolymers were calculated based on the integration areas of the characteristic peaks of PEO and polyester (P(CL-*ran*-VL)).

### 3.2. Nanoparticle Preparation—DLS Analysis

**Figure 2:**
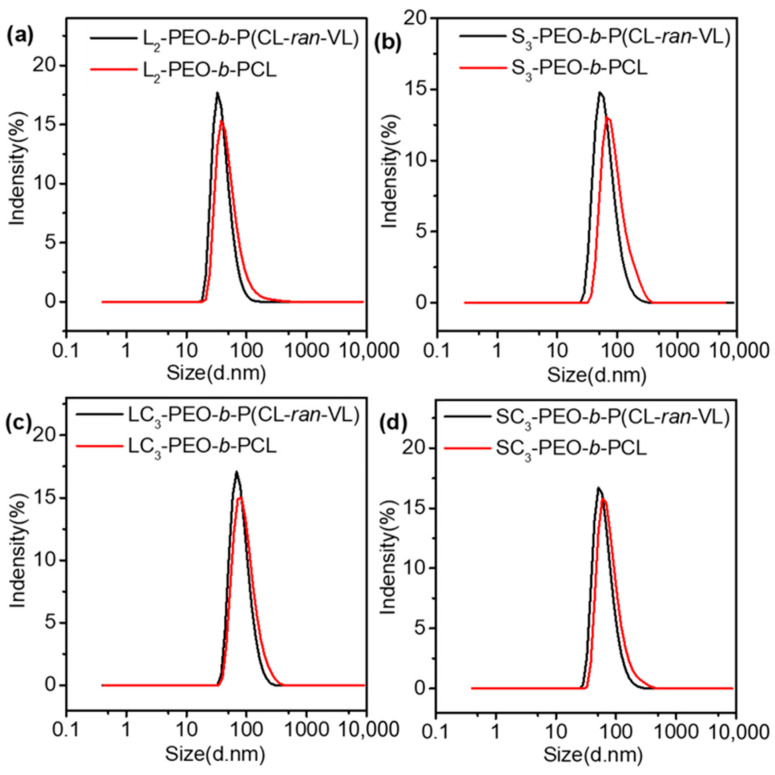
DLS curves of PEO-*b*-P(CL-*ran*-VL) and PEO-*b*-PCL with different architectures: (**a**) L_2_-PEO-*b*-P(CL-*ran*-VL) and L_2_-PEO-*b*-PCL, (**b**) S_3_-PEO-*b*-P(CL-*ran*-VL) and S_3_-PEO-*b*-PCL, (**c**) LC_3_-PEO-*b*-P(CL-*ran*-VL) and LC_3_-PEO-*b*-PCL, (**d**) SC_3_-PEO-*b*-P(CL-*ran*-VL) and SC_3_-PEO-*b*-PCL.

The hydrodynamic diameters (D_h_) of all micelles (before and after drug loading) were below 200 nm, falling within the optimal size range for prolonged circulation in vivo [18,49,50]. TEM analysis of the micelle morphology revealed that each copolymer micelle exhibited a uniformly distributed spherical shape (Figure S2). As shown in Figure 2 and Table 2, all PEO-*b*-P(CL-*ran*-VL) copolymers demonstrated monomodal size distribution curves, and the PDI of all micelles was less than 0.3, indicating that the micelle morphology was consistent and the size was uniform.

**Figure 3:**
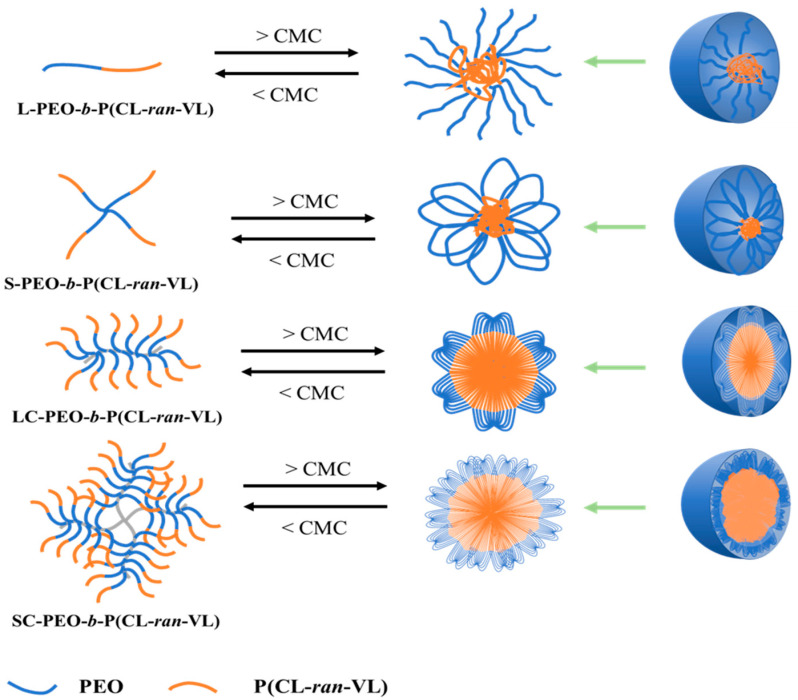
Schematic diagram of the flower micelle model.

In terms of copolymer composition, as the side chain length of the linear copolymer increased (from L_1_-PEO-*b*-P(CL-*ran*-VL) to L_3_-PEO-*b*-P(CL-*ran*-VL)), the micelle size increased from 42 to 87.3 nm (Table 2). Linear copolymers form micelles in water through the self-assembly of multiple chains. When the concentration of linear copolymers in the solution exceeds the CMC, the hydrophobic blocks of the polymer assemble to form a core, while the hydrophilic blocks extend outward to form a hydrophilic shell (Figure 3) [51]. The increase in molecular weight from 1.8 kg/mol to 8.1 kg/mol (Table 1) and the consequent elongation of the hydrophilic and hydrophobic blocks resulted in an expanded micelle core volume and a thicker hydrophilic shell, thereby increasing the micelle size.

However, the star copolymers S-PEO-*b*-P(CL-*ran*-VL) and highly branched copolymers LC-/SC-PEO-*b*-P(CL-*ran*-VL) showed an opposite trend, where the micelle size decreased with increasing size as the complexity of the molecular architecture increased, and this trend was further strengthened. The micelle size of S_3_-PEO-*b*-P(CL-*ran*-VL) was 18.2 nm smaller than that of S_1_-PEO-*b*-P(CL-*ran*-VL), and the size of SC_3_-PEO-*b*-P(CL-*ran*-VL) decreased by 46.7 nm compared to SC_1_-PEO-*b*-P(CL-*ran*-VL) (Table 2). The main reason for this phenomenon was the different self-assembly processes of the branched copolymers in water. Before explaining this phenomenon, it is crucial to describe the procedure of micelle assembly from branched amphiphilic copolymers.

For branched amphiphilic copolymers, including S-PEO-*b*-P(CL-*ran*-VL), LC-PEO-*b*-P(CL-*ran*-VL), and SC-PEO-*b*-P(CL-*ran*-VL), when the hydrophilic head faced inside and the hydrophobic tail projected outside, the hydrophobic PET block needed to be bent, and the hydrophilic PEO block had to be exposed on the outside to wrap the hydrophobic block inside to form micelles (Figure 3). In micelles assembled through this mechanism, each copolymer molecule contains a compact internal space that provides significant drug-loading capacity [52,53,54].Owing to the compact structure of the branched copolymers, the resisting forces between the side chains were substantial, and the side chain mobility was limited by the primary chain. When the side chains were short, the copolymers exhibited greater rigidity, making it difficult for the outer PEO blocks to bend into micelle cores. Consequently, more copolymer molecules were required to form micelles, resulting in larger particle sizes. In contrast, as the side-chain length increased, the polymer chain became more flexible and bent more easily. This caused the micelles formed by the highly branched copolymers to shrink.

### 3.3. Self-Assembly (CMC Determination)

Upon reaching the CMC, PEO-*b*-P(CL-*ran*-VL) began to self-assemble, producing thermodynamically stable structures. CMC was the minimum concentration required to characterize the stability of drug carriers [17]. The intensity ratio of the pyrene excitation spectra increased with increasing polarity. The micelle formation process was observed by monitoring the variations in the spectral signals of the pyrene probe, and the CMC was determined.

**Figure 4:**
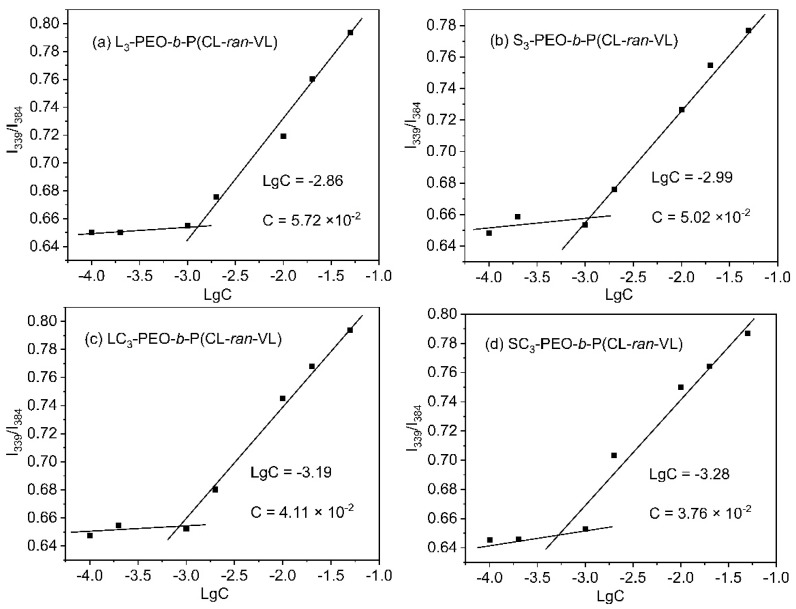
Plots of the intensity ratio (I339/I384) against the concentrations of (**a**) L_3_-PEO-*b*-P(CL-*ran*-VL), (**b**) S_3_-PEO-*b*-P(CL-*ran*-VL), (**c**) LC_3_-PEO-*b*-P(CL-*ran*-VL), and (**d**) SC_3_-PEO-*b*-P(CL-*ran*-VL).

The impact of polymer architecture on the thermal stability of the self-assembled micelles was further explored. CMC as a key parameter for micelle stability, and the CMC values for L_3_-PEO-*b*-P(CL-*ran*-VL) was 5.72 × 10^−2^ mg/mL (Figure 4a) and S_3_-PEO-*b*-P(CL-*ran*-VL) was 5.02 × 10^−2^ mg/mL (Figure 4b), respectively. Similar values were obtained for the linear- and star-shaped copolymers, indicating that these two molecular architectures were not key factors affecting micelle stability. Furthermore, all micelles exhibited a trend of decreasing CMC with an increase in molecular weight (Table S2). In amphiphilic block copolymers, the CMC was found to decrease as the size of hydrophobic blocks increased. This was attributed to the enhanced hydrophobic interactions, which promoted micelle formation at lower concentrations. The expansion of hydrophilic blocks was observed to improve the solubility of copolymers in water, potentially leading to a slight increase in CMC. When both hydrophobic and hydrophilic blocks were increased simultaneously, hydrophobic interactions were found to dominate, resulting in a decrease in CMC [55].

However, when the copolymer structure appeared as a ‘comb’, the CMC value changed significantly. The CMC for LC_3_-PEO-*b*-P(CL-*ran*-VL) was 4.11 × 10^−2^ mg/mL (Figure 4c), and that for SC_3_-PEO-*b*-P(CL-*ran*-VL) was 3.76 × 10^−2^ mg/mL (Figure 4d). Comparing linear and linear–comb or star and star–comb copolymers showed a significant decrease in the CMC. High-density side chains increase the length and degree of hydrophilicity of hydrophilic chains, reduce aggregation and fusion between micelles, and make micelles more stable [55]. Therefore, comb structures play a crucial role in influencing the self-assembly behavior in the design of molecular structures.

### 3.4. Stability and Drug Encapsulation

Copolymer drug-loaded micelles with different architectures were prepared at optimal drug loading (Figure S3) to investigate their encapsulation efficiency and drug loading capacity.

Table 3 provides a summary of the DL (%) and EE (%) values. SC_3_-PEO-*b*-P(CL-*ran*-VL) exhibited the highest values of DL (%) and EE (%), with a DL of 8.2% and an EE of 39.8%. Compared to polymers with similar molecular architectures but different side chain lengths, this architecture was optimal for curcumin encapsulation. The DL (%) and EE (%) values for SC_1_-PEO-*b*-P(CL-*ran*-VL) were 5.5% and 27.3%, respectively. Similar trends were observed for the copolymers with other architectures. For each copolymer with the same architecture, where the hydrophilic and hydrophobic contents of each molecule were nearly identical, a longer side chain length correlated with improved encapsulation. The increase in side chain molecular weight enhanced the encapsulation capacity of the micelles, facilitating greater drug loading.

On the one hand, L_3_-PEO-*b*-P(CL-*ran*-VL) and S_3_-PEO-*b*-P(CL-*ran*-VL) also showed relatively high DL and EE, with DL% and EE% values similar to those of DL (%) and EE (%) of L_3_-PEO-*b*-P(CL-*ran*-VL) being 7.0% and 34.8%, respectively, and the DL (%) and EE (%) values for S_3_-PEO-*b*-P(CL-*ran*-VL) are recorded at 6.5% and 32.4%, respectively. The encapsulation efficiency showed little variation, regardless of whether the polymer was linear or star-shaped.

In contrast, the hyperbranched copolymer (LC-/SC-PEO-*b*-P(CL-*ran*-VL)) exhibited higher encapsulation values than the linear and star copolymers. EE% and DL% increased as the branching degree increased, and the drug loading and encapsulation efficiency increased from 4.9% and 24.3% for S_1_-PEO-*b*-P(CL-*ran*-VL) to 5.5% and 27.3% for SC_1_-PEO-*b*-P(CL-*ran*-VL) copolymers. The linear and comb polymers exhibited similar trends. These results demonstrate that the branched architectures of copolymers can significantly improve the drug loading efficiency. This may be due to the high density of the side chains, which increases the packing density of the hydrophobic chains, creating a tighter core that can encapsulate more drugs. Meanwhile, the dense arrangement of side chains enhances steric hindrance, prevents drug leakage, and improves encapsulation efficiency.

**Figure 5:**
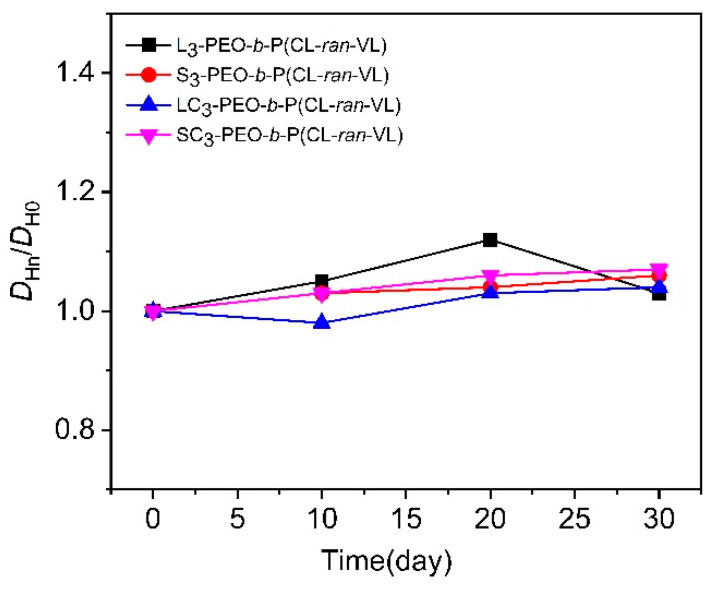
Stability study of polymeric micelles.

The examination also focused on how drug encapsulation affects particle size, and the size distributions were recorded over a 30-day period. After the copolymer micelles were prepared, the micelle solution was periodically subjected to DLS testing at room temperature. These changes are shown in Figure 5. The particle size of the copolymer micelles increased slightly over time, but the change was weak. This may be due to the increased hydrophilicity of the hydrophobic core after drug release, water molecule infiltration, and micelle swelling, which enlarges the particle size. For the linear copolymers, the micelle particle size notably decreased from days 20 to 30. This may be due to the structure of linear copolymers. In contrast to hyperbranched copolymers, linear copolymers form micelles without a denser core and thick shell. A faster drug release rate could lead to partial micelle disintegration as the hydrophobic core diminishes, thus reducing the particle size.

**Figure 6:**
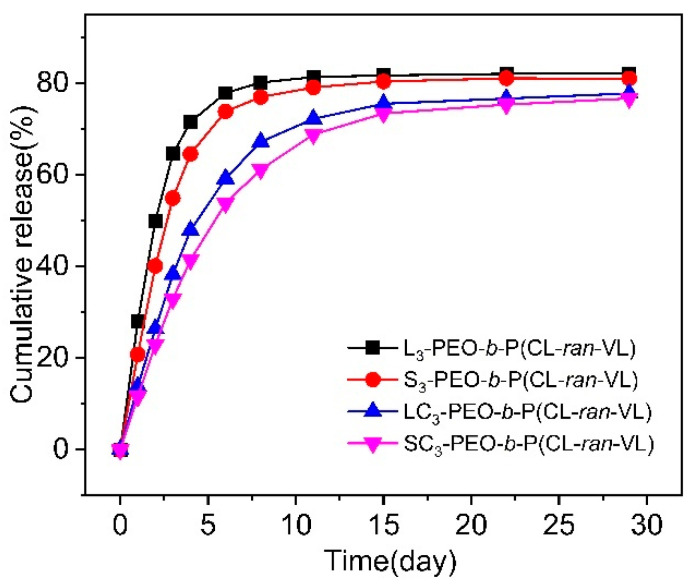
Cumulative release curves of curcumin-loaded micelles with different architectures for in vitro drug release.

To tailor drug release for various bio-applications, the in vitro release of curcumin encapsulated in L-/S-/LC-/SC-PEO-*b*-P(CL-*ran*-VL) was examined using PBS. A swift release of 70% of curcumin occurred within the initial 7 days, which then transitioned to a more gradual release over the subsequent 23 days. This early rapid release is likely due to curcumin diffusion through the hydrophobic core of the P(CL-*ran*-VL) micelles (Figure 6). At this stage, the release rate of curcumin decreased as the branching of the PEO-*b*-P(CL-*ran*-VL) copolymers increased, probably because more highly branched copolymers created a denser core. Owing to the suitable micelle size and high drug-loading efficiency of SC-PEO-*b*-P(CL-*ran*-VL), this copolymer has promising application prospects in tumor therapy. It should be emphasized that this rapid release was defined in relation to the subsequent release rates. Indeed, in numerous drug-loaded micelles, upon placement in the release medium, an initial large-dose drug was immediately released before the release rate attained the steady-state curve, a phenomenon commonly referred to as ‘burst release’ [56]; the micelles in this study did not demonstrate burst release.

### 3.5. In Vitro Cytotoxicity

**Figure 7:**
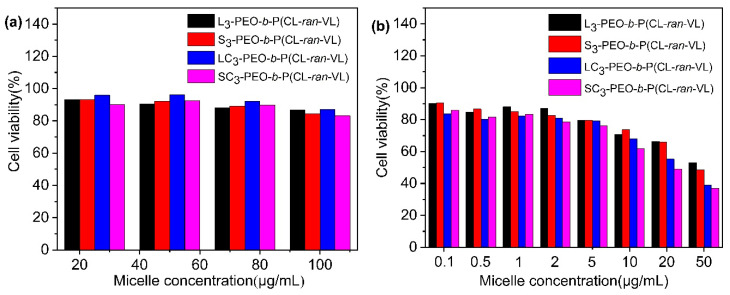
PANC-1 cell viability after 48 h by CCK-8 assay (*n* = 3): (**a**) blank micelles and (**b**) drug-loaded micelles.

The CCK-8 assay was used to assess cell viability with and without micelles loaded with curcumin. Figure 7a illustrates the blank micelles in vitro after 48 h, with a cell viability above 80%. The influence of micelle concentration on cell viability was minimal, and no or low toxicity was observed. All the copolymers exhibited excellent biocompatibility. Substantially reduced cell viability was observed after incubation with curcumin-loaded micelles under the same conditions. This suggests that micelles containing curcumin and composed of PEO-*b*-P(CL-*ran*-VL) effectively suppressed PANC-1 cells. When the concentration reached 2 μg/mL, the cell viability dropped to below 60%. An increase in micelle concentration led to increased cytotoxicity, as shown in Figure 7b. Furthermore, the suppressive effect was concentration dependent.

A comparative examination of micelles with varying topological architectures revealed that the degree of cancer cell inhibition increased with the degree of branching (micelle concentrations above 2 μg/mL). In summary, SC-PEO-*b*-P(CL-*ran*-VL) micelles exhibited a superior drug-loading capacity, controlled release behavior, selective cytotoxicity against tumor cells, and potential for cancer therapy.

## 4. Conclusions

A range of precisely structured linear–comb and star–comb PEO-*b*-P(CL-*ran*-VL) materials were effectively synthesized via ring-opening polymerization (ROP). A library of linear, star, linear–comb, and star–comb block copolymers with predetermined side chain lengths, degrees of branching, and narrow dispersions was obtained using L-PB-OH and S-PB-OH initiators. This study indicates that micelles with complex structures demonstrate enhanced stability while maintaining a constant side chain length. Furthermore, in addition to linear copolymers, an increase in the side chain length facilitated the formation of smaller micelles. Additionally, the stability, encapsulation performance, cytotoxicity, and self-assembly of drug micelles were investigated. In drug delivery applications, star–comb copolymers (SC-PEO-*b*-P(CL-*ran*-VL)) with P(CL-*ran*-VL) cores and PEO shells exhibit an optimal combination of small particle size, stability, and relatively high drug loading capacity. The results emphasize the importance of the degree of polymer branching and side-chain length in the design of drug delivery nanocarriers. The results suggest that this novel star-shaped comb-like block copolymer exhibits superior stability and drug-loading capacity, rendering it a potentially optimal drug carrier.

## Figures and Tables

**Table 1:** Synthesis and characterization of PEO and PEO-*b*-P(CL-*ran*-VL) with different topological architectures.

Sample	[OH] ^a^	[EO]/[OH] ^b^	PEO Block			Total			PEO/PET ^i^	PEO/PET ^j^	Yield ^k^ (%)
			M_n_^c^ (kg/mol)	M_n_^d^ (kg/mol)	PDI ^e^	M_n_^f^ (kg/mol)	M_n_^g^ (kg/mol)	PDI ^h^			
L_1_-PEO-*b*-P(CL-*ran*-VL)	-	40	0.9	-	1.07	1.8	-	1.18	1.00	0.87	74
L_2_-PEO-*b*-P(CL-*ran*-VL)	-	80	1.9	-	1.09	3.7	-	1.18	1.05	0.87	80
L_3_-PEO-*b*-P(CL-*ran*-VL)	-	150	4.0	-	1.10	8.1	-	1.09	0.98	0.92	85
S_1_-PEO-*b*-P(CL-*ran*-VL)	-	30	3.7	0.94	1.05	6.4	1.36	1.21	1.37	0.92	94
S_2_-PEO-*b*-P(CL-*ran*-VL)	-	50	5.4	1.35	1.08	9.6	2.42	1.16	1.29	0.89	93
S_3_-PEO-*b*-P(CL-*ran*-VL)	-	70	7.4	1.85	1.11	13.4	3.36	1.21	1.35	0.84	89
LC_1_-PEO-*b*-P(CL-*ran*-VL)	23	40	19.6	0.85	1.14	34.3	1.49	1.16	1.33	0.79	85
LC_2_-PEO-*b*-P(CL-*ran*-VL)	23	50	30.4	1.32	1.12	51.4	2.24	1.13	1.44	0.92	87
LC_3_-PEO-*b*-P(CL-*ran*-VL)	23	60	43.2	1.88	1.06	75.7	3.29	1.21	1.32	0.94	83
SC_1_-PEO-*b*-P(CL-*ran*-VL)	23	40	22.3	1.01	1.14	40.1	1.82	1.13	1.25	0.83	77
SC_2_-PEO-*b*-P(CL-*ran*-VL)	23	50	34.7	1.58	1.15	58.1	2.64	1.25	1.48	0.84	81
SC_3_-PEO-*b*-P(CL-*ran*-VL)	23	60	41.6	1.89	1.10	72.8	3.31	1.21	1.33	0.98	79

^a^ The average number of hydroxide groups in the side chains was determined using NMR calculations. ^b^ Molar ratio of ethylene oxide and hydroxide groups in the feed solution. ^c^ The PEO content was determined using GPC. ^d^ Determination of single-arm average molecular weight of PEO by NMR calculation. ^e^ The dispersity of PEO was determined by GPC. ^f^ Measured by GPC after connecting the polyester blocks. ^g^ Determination of the single-arm average molecular weight after blocking the polyester blocks. ^h^ The dispersity of PEO-*b*-P(CL-*ran*-VL) was determined using GPC. ^i^ The ratio of the molecular weights of PEO to P(CL-*ran*-VL) was determined using GPC. ^j^ The ratio of the molecular weights of PEO to P(CL-*ran*-VL) was determined using ^1^H NMR spectroscopy. ^k^ Yield of PEO-b-P(CL-ran-VL) with different architectures.

**Table 2:** DLS parameters of PEO-*b*-P(CL-*ran*-VL) with different architectures.

Sample	*D*_H_ ^(1)^ (nm)	PDI ^(1)^	*D*_H_-_Cur_ ^(2)^ (nm)	PDI ^(2)^
L_1_-PEO-*b*-(CL-*ran*-VL)	42.0 ± 1	0.12 ± 0.04	41.7 ± 1	0.13 ± 0.02
L_2_-PEO-*b*-(CL-*ran*-VL)	54.6 ± 1	0.23 ± 0.01	50.2 ± 1	0.24 ± 0.01
L_3_-PEO-*b*-(CL-*ran*-VL)	87.3 ± 1	0.13 ± 0.01	76.4 ± 1	0.16 ± 0.03
S_1_-PEO-*b*-(CL-*ran*-VL)	128.8 ± 1	0.10 ± 0.01	106.3 ± 1	0.13 ± 0.01
S_2_-PEO-*b*-(CL-*ran*-VL)	117.4 ± 1	0.19 ± 0.02	94.2 ± 1	0.21 ± 0.01
S_3_-PEO-*b*-(CL-*ran*-VL)	110.6 ± 1	0.16 ± 0.01	93.2 ± 1	0.17 ± 0.02
LC_1_-PEO-*b*-(CL-*ran*-VL)	128.6 ± 1	0.23 ± 0.03	107.2 ± 1	0.26 ± 0.01
LC_2_-PEO-*b*-(CL-*ran*-VL)	117.0 ± 1	0.18 ± 0.01	87.7 ± 1	0.19 ± 0.01
LC_3_-PEO-*b*-(CL-*ran*-VL)	102.8 ± 1	0.11 ± 0.01	92.4 ± 1	0.14 ± 0.01
SC_1_-PEO-*b*-(CL-*ran*-VL)	143.5 ± 1	0.12 ± 0.01	125.3 ± 1	0.16 ± 0.01
SC_2_-PEO-*b*-(CL-*ran*-VL)	118.7 ± 1	0.12 ± 0.03	118.2 ± 1	0.13 ± 0.01
SC_3_-PEO-*b*-(CL-*ran*-VL)	96.8 ± 1	0.17 ± 0.01	100.2 ± 1	0.19 ± 0.01

^(1)^ Blank micelle DLS parameters: ^(2)^ DLS parameters of drug-loaded micelles.

**Table 3:** Delivery performance parameters of PEO-*b*-P(CL-*ran*-VL) with different architectures.

Sample	DL (%)	EE (%)
L_1_-PEO-*b*-(CL-*ran*-VL)	4.5 ± 0.5	22.3 ± 1
L_2_-PEO-*b*-(CL-*ran*-VL)	5.5 ± 0.2	27.5 ± 1
L_3_-PEO-*b*-(CL-*ran*-VL)	7.0 ± 1	34.8 ± 2
S_1_-PEO-*b*-(CL-*ran*-VL)	4.9 ± 1	24.3 ± 1
S_2_-PEO-*b*-(CL-*ran*-VL)	5.7 ± 0.5	28.6 ± 1
S_3_-PEO-*b*-(CL-*ran*-VL)	6.5 ± 1	32.4 ± 2
LC_1_-PEO-*b*-(CL-*ran*-VL)	5.0 ± 1	25.1 ± 1
LC_2_-PEO-*b*-(CL-*ran*-VL)	6.1 ± 1	30.4 ± 1
LC_3_-PEO-*b*-(CL-*ran*-VL)	7.7 ± 1	38.5 ± 1
SC_1_-PEO-*b*-(CL-*ran*-VL)	5.5 ± 1	27.3 ± 1
SC_2_-PEO-*b*-(CL-*ran*-VL)	6.6 ± 1	33.1 ± 3
SC_3_-PEO-*b*-(CL-*ran*-VL)	8.2 ± 1	39.8 ± 1

## Data Availability

The original contributions presented in the study are included in the article/Supplementary Materials; further inquiries can be directed to the corresponding author.

## Supplementary Materials

The following supporting information can be downloaded at: https://www.mdpi.com/article/10.3390/polym17081030/s1, Table S1. Molecular data of linear/star hydroxylated poly(1,4-butadiene); Table S2. The critical micelle concentration of copolymers with different architectures; Figure S1. The standard curve equation was y = 0.123x + 0.006, R^2^ = 0.99995. Standard curve of curcumin in PBS/acetone (4:1, *v*/*v*): (a) UV-Spectroscopic spectrum. (b) Linearity plot; Figure S2. TEM images of PEO-*b*-P(CL-*ran*-VL) with different architectures: (a) L_1_-PEO-*b*-P(CL-*ran*-VL); (b) S_2_-PEO-*b*-P(CL-*ran*-VL); (c) LC_2_-PEO-*b*-P(CL-*ran*-VL); (d) SC_2_-PEO-*b*-P(CL-*ran*-VL); Figure S3. Condition of micelles prepared using different dosages: (a) initial condition. (b) After 10 days.
