# Supplementary material for: Study of Polyethylene Oxide-b-Poly(ε-caprolactone-ran-δ-valerolactone) Amphiphilic Architectures and Their Effects on Self-Assembly as a Drug Carrier
Source: Polymers (Basel). 2025 Apr 10;17(8):1030. doi: 10.3390/polym17081030 (PMC12030258; doi:10.3390/polym17081030)
Supplement: Supplementary file 1 [file polymers-17-01030-s001.zip › polymers-3554949-supplementary.pdf]

Supporting Information

Study of polyethylene oxide-*b*- poly(*ε*-caprolactone-*ran*-*δ*-valerolactone) amphiphilic architectures and their effects on self-assembly as a drug carrier

Chaoqun Wang [a 1], Tong Wu [b 1], Yidi Li[a], Jie Liu[a], Yanshai Wang[a], Kefeng Wang[a], Yang Li [a] *, Xuefei Leng [a] *

[a] *State Key Laboratory of Fine Chemicals, Department of Polymer Science and Engineering, Liaoning Key Laboratory of Polymer Science and Engineering, School of Chemical Engineering, Dalian University of Technology, Dalian 116024 China.*

[b] *SINOPEC Ningbo New Materials Research Institute Company Limited, Ningbo, China.*

[1] *These authors contributed equally to this work.*

Corresponding authors:

*E-mail: liyang@dlut.edu.cn (Y. Li)

*E-mail: lengxuefei@dlut.edu.cn (X. Leng)

Synthesis of Polybutadiene

All synthesis procedures were conducted under a nitrogen atmosphere. Initially, cyclohexane and a THF regulator (3.6 mmol) were introduced into a Schlenk flask that had been subjected to three cycles of vacuum evacuation, heating, and nitrogen purging. An appropriate quantity of butadiene (5.0 g, 185.2 mmol) was vaporized in a cyclohexane solution (10 wt%). Subsequently, the polymerization vessel was immersed in a constant-temperature water bath maintained at 50 °C for 30 minutes. Following the stabilization of temperature, n-butyl lithium (5.2 mmol) was added, and the reaction was allowed to proceed at 50 °C for 3 hours. The reaction was terminated by the addition of excess isopropanol. The resulting colorless and transparent linear polybutadiene (L-PB) was washed with water until neutral and then dried under vacuum at 40 °C to achieve a constant weight.

The synthesis procedure for star polybutadiene (S-PB) closely resembles that of linear polybutadiene (L-PB). The polymerization of butadiene is conducted over a duration of 3 hours. Prior to the introduction of the isopropanol termination agent, a precise quantity of the $SiCl_4$ coupling agent is incorporated. The coupling reaction is executed at a temperature of 50 °C. Following 5 hours of reaction, an excess of the terminating agent is introduced to yield four-arm star-shaped polybutadiene (S-PB).

Table S1. Molecular data of linear/star hydroxylated poly(1,4-butadiene)

Initiator	Mn[a] (kDa)	Mn[b] (kDa)	Mn[c] (kDa)	PDI[c]	[OH][d]	yield[e] (%)
$L\text{-}PB\text{-}OH_1$	4.0	4.9	8.5	1.15	23	98
$S\text{-}PB\text{-}OH_1$	4.0	5.5	8.8	1.24	23	96

[a] Designed molecular weight. [b] Determined by NMR spectroscopy. [c] Determined by GPC using THF as the eluent and PS as the standard. [d] Number of hydroxide groups determined by NMR calculation. [e] Calculated from the weight of the monomer and product.

Curcumin UV absorption standard curve.

Curcumin (50.0 mg) was precisely weighed and placed into a 100 mL volumetric flask, where it was dissolved in a PBS/acetone mixture (v/v=4/1) and subsequently diluted to 100 ml. A 1.0 mL aliquot of this solution was then transferred into a 50 mL

volumetric flask using a pipette and further diluted with the same PBS/acetone mixture to achieve a curcumin concentration of 0.01 mg/mL. Subsequently, aliquots of 0.5 mL, 1.0 mL, 2.0 mL, 3.0 mL, 4.0 mL, 5.0 mL, and 6.0 mL of the aforementioned solution were transferred into separate 10 mL volumetric flasks and diluted with the PBS/acetone mixture (v/v=4/1) to prepare curcumin standard solutions with concentrations ranging from 0.5 μg/mL to 6.0 μg/mL. The UV absorption spectra of each solution were measured using a UV-visible spectrophotometer, with the absorption peak at 427 nm being recorded. A graph depicting the relationship between curcumin concentration and absorption peak was constructed and subjected to linear fitting to derive the UV absorption standard curve of curcumin. Standard curve as illustrated in

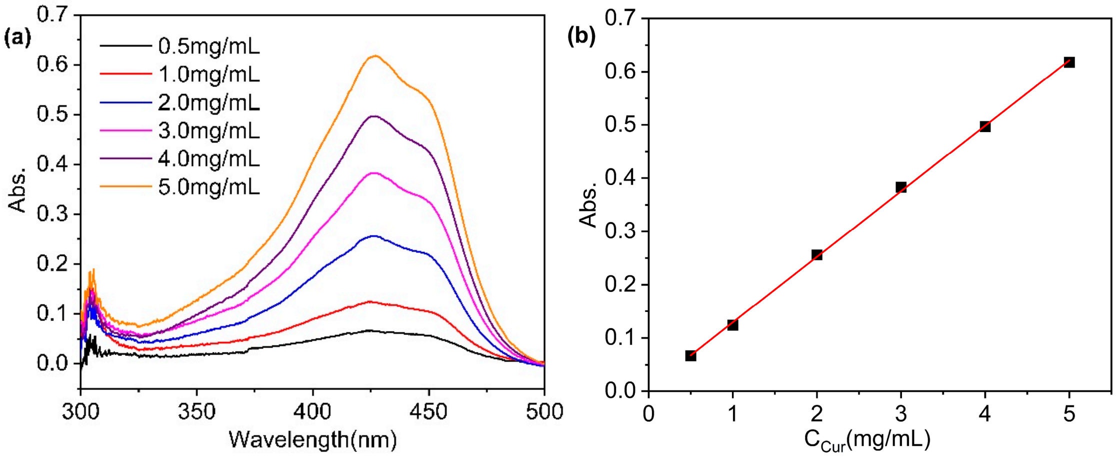

Figure S1. The standard curve equation was y = 0.123x + 0.006, R^2 = 0.99995. Standard curve of curcumin in PBS/acetone (4:1, v/v): (a) UV-Spectroscopic spectrum. (b) Linearity plot.

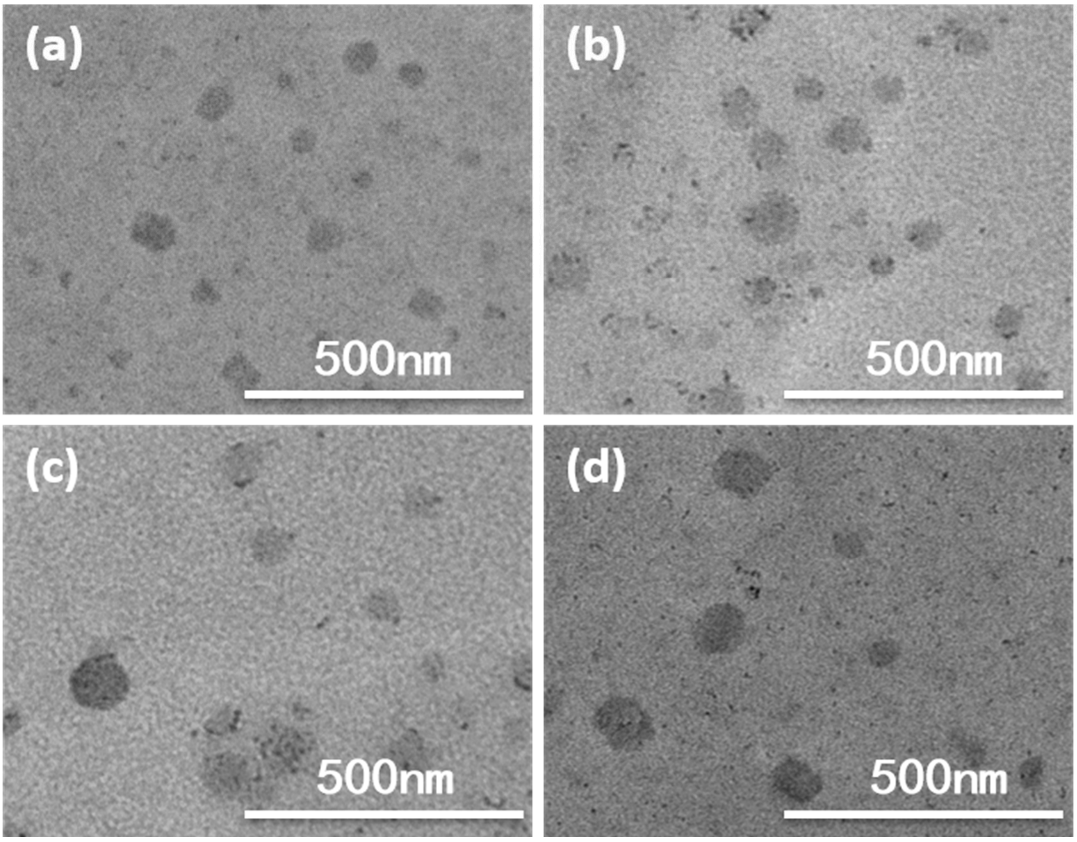

Figure S2. TEM images of PEO-*b*-P(CL-*ran*-VL) with different architectures: (a) L_1-PEO-*b*-P(CL-*ran*-VL); (b) S_2-PEO-*b*-P(CL-*ran*-VL); (c) LC_2-PEO-*b*-P(CL-*ran*-VL); (d) SC_2-PEO-*b*-P(CL-*ran*-VL).

Table S2. The critical micelle concentration of copolymers with different architectures.

Sample	CMC(mg/ml)
L_1-PEO-*b*-(CL-*ran*-VL)	$7.57\times10^{-2}\pm0.003$
L_2-PEO-*b*-(CL-*ran*-VL)	$6.81\times10^{-2}\pm0.002$
L_3-PEO-*b*-(CL-*ran*-VL)	$5.72\times10^{-2}\pm0.003$
S_1-PEO-*b*-(CL-*ran*-VL)	$7.11\times10^{-2}\pm0.001$
S_2-PEO-*b*-(CL-*ran*-VL)	$6.58\times10^{-2}\pm0.003$
S_3-PEO-*b*-(CL-*ran*-VL)	$5.02\times10^{-2}\pm0.004$
LC_1-PEO-*b*-(CL-*ran*-VL)	$6.14\times10^{-2}\pm0.002$
LC_2-PEO-*b*-(CL-*ran*-VL)	$5.36\times10^{-2}\pm0.002$
LC_3-PEO-*b*-(CL-*ran*-VL)	$4.11\times10^{-2}\pm0.003$
SC_1-PEO-*b*-(CL-*ran*-VL)	$5.47\times10^{-2}\pm0.001$
SC_2-PEO-*b*-(CL-*ran*-VL)	$4.53\times10^{-2}\pm0.003$
SC_3-PEO-*b*-(CL-*ran*-VL)	$3.76\times10^{-2}\pm0.003$

To ascertain the optimal drug loading for amphiphilic polymer micelles with varying topological structures, a linear comb amphiphilic polymer, LC_3-PEO-*b*-P(CL-

ran-VL), was employed as a model. Drug-loaded micelles were prepared using polymer-drug ratios of 10:1, 5:1, 3:1, and 2:1 and were subsequently monitored. As illustrated in Figure S3(a), the micelles remained clear and transparent at drug loading ratios of 10:1, 5:1, and 3:1, with the colour intensifying as the drug loading ratio increased. At a 2:1 ratio, the drug-loaded micelles exhibited turbidity owing to the aggregation of unencapsulated drug particles in water.

Upon storage, the samples displayed different states (Figure S3(b)). Although all drug-loaded micelles appeared clear, those at ratios of 3:1 and 2:1 demonstrated flocculent precipitates at the bottom of the container, indicating poor stability. Consequently, a drug loading ratio of 5:1 was selected for subsequent drug loading and in vitro release experiments.

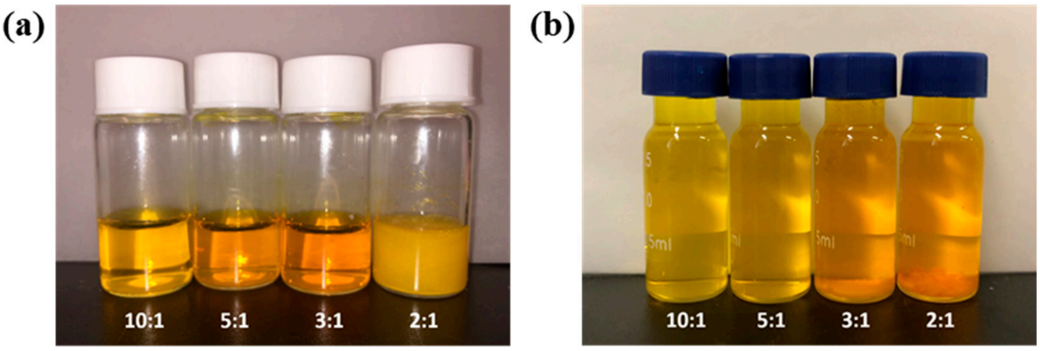

Figure S3. Condition of micelles prepared using different dosages: (a) initial condition. (b) After 10 days.
